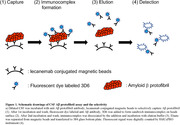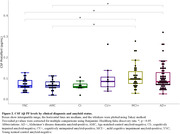# Lecanemab‐associated amyloid‐β protofibril is a proximal biomarker of neurodegeneration unlike other plaque‐associated biomarkers

**DOI:** 10.1002/alz.094585

**Published:** 2025-01-09

**Authors:** Kenjiro Ono, Moeko Shinohara, Kazuyoshi Shuta, Hidetomo Murakami, Yukiko Mori, Junji Komatsu, Chizuru Kobayashi, Steven Hersch, Kanta Horie

**Affiliations:** ^1^ Kanazawa University Graduate School of Medical Sciences, Kanazawa Japan; ^2^ Deep Human Biology Learning (DHBL), Eisai Co., Ltd., Tsukuba Japan; ^3^ School of Medicine, Showa University, Tokyo Japan; ^4^ Deep Human Biology Learning (DHBL), Eisai Inc., Nutley, NJ USA

## Abstract

**Background:**

We developed a highly sensitive immunoassay method using lecanemab that selectively captures amyloid‐β (Aβ) protofibril (PF). To characterize Aβ‐PF in human cerebrospinal fluid (CSF), we investigated the CSF Aβ‐PF levels in patients with Alzheimer’s disease (AD) at different disease stages. We also studied the association of CSF Aβ‐PF with other AD‐related biomarkers.

**Method:**

The participants in this study consisted of 48 cognitively unimpaired Aβ‐negative (CU‐) including 25 young normal control (YNC) and 23 age matched control (AMC) (age≥50), 8 cognitively impaired diagnosed as suspected non‐Alzheimer’s pathophysiology (CI‐), 9 cognitively unimpaired Aβ‐positive (CU+), 34 Aβ‐positive with mild cognitive impairment (MCI+) and 64 Aβ‐positive with AD dementia (AD+). CSF Aβ‐PF were analyzed by the immunoassay system using lecanemab as a tool antibody. The other CSF biomarkers were analyzed by the conventional enzyme‐linked immunosorbent assay (ELISA) or the digital ELISA system (Simoa) (Figure 1).

**Result:**

The CSF Aβ‐PF levels significantly increased in MCI+ and AD+ compared to YNC or AMC in CU‐ group (Figure 2). CU+ also showed increase of CSF Aβ‐PF levels while CI‐ did not although the differences were not significant. In Aβ‐positive participants, the CSF Aβ‐PF was strongly correlated with “N” biomarkers such as CSF total tau (t‐tau) (Spearman rho = 0.634, p < 0.001) and neurogranin (rho = 0.434, p < 0.001) whereas the relatively lower correlations were observed for CSF Aβ42 (rho = ‐0.080, p = 0.415), Aβ42/40 (rho = ‐0.334, p < 0.001), tau phosphorylated at 181 residue (p‐tau181) (rho = 0.294, p = 0.004), and p‐tau217 (rho = 0.232, p = 0.018) which are known as plaque‐associated biomarkers.

**Conclusion:**

This is the first report describing lecanemab‐captured Aβ‐PF species in human CSF. The CSF Aβ‐PF levels increased during AD continuum including MCI+ as well as AD+ stages. CSF Aβ‐PF showed modest correlation with plaque‐associated biomarkers in Aβ‐positive participants and stronger correlation with neurodegeneration biomarkers, suggesting that lecanemab‐associated Aβ‐PF may be a toxic species involved in the amyloid cascade and underlie the clinical effect of lecanemab in AD.